# Predictive Validity of Multifactorial Injury Risk Models and Associated Clinical Measures in the U.S. Population

**DOI:** 10.3390/sports12050123

**Published:** 2024-04-28

**Authors:** Adam C. Eckart, Pragya Sharma Ghimire, James Stavitz

**Affiliations:** 1 Department of Health and Human Performance, College of Health Professions and Human Services, Kean University, 1000 Morris Avenue, Union, NJ 07083, USA; pghimire@kean.edu; 2 Department of Athletic Training, College of Health Professions and Human Services, Kean University, 1000 Morris Avenue, Union, NJ 07083, USA; jstavitz@kean.edu

**Keywords:** musculoskeletal injuries, injury risk factors, pain, physical functioning, bone turnover markers, inflammation

## Abstract

**Background:** Popular movement-based injury risk screens were shown to lack predictive precision, leading to interest in multifactorial models. Furthermore, there is a lack of research regarding injury risk assessment for those currently or planning to be recreationally active. This study aims to provide injury risk insights by analyzing multifactorial injury risk models and associated clinical measures in the U.S. population. **Methods:** Data related to injury, inflammatory markers, physical functioning, body composition, physical activity, and other variables from 21,033 respondents were extracted from NHANES. Odds ratios for self-reported injury were calculated for single predictors and risk models. Case–control and principal component analyses (PCA) were conducted to elucidate confounders and identify risk factor clusters, respectively. Receiver operating characteristic analysis was used to test the precision of a risk factor cluster to identify pain points and functional difficulties. **Results:** Sociodemographic, individual, and lifestyle factors were strongly associated with higher odds of injury. Increases in fibrinogen and C-reactive protein were significantly associated with all risk groups. Membership to the high-risk group (age over 40, obesity, no muscle-strengthening activities, sedentary lifestyle, and low back pain) predicted at least one functional difficulty with 67.4% sensitivity and 87.2% specificity. In the injury group, bone turnover markers were higher, yet confounded by age, and there was a significantly higher prevalence of self-reported osteoporosis compared to the control. In males, low testosterone was associated with injury, and high estradiol was associated with pain and functional difficulties. In females, high follicle-stimulating hormone was associated with functional difficulties. PCA revealed four high-risk profiles, with markers and activities showing distinct loadings. **Conclusions:** A comprehensive approach to injury risk assessment should consider the nexus of aging, lifestyle, and chronic disease to enhance tailored injury prevention strategies, fostering safe and effective physical activity participation and reducing the burden of musculoskeletal disorders.

## 1. Introduction

Musculoskeletal disorders affect one in two U.S. adults and are a leading cause of years lived with disability [1]. Physical inactivity increases the risk of musculoskeletal disorders and early mortality [1]. Conversely, there is a dose–response relationship between physical workloads and risk of musculoskeletal injuries (MSIs) [2]. Moreover, MSI risk was deemed proportional to the gap between an individual’s habitual physical activity (PA) level and their current PA level [2]. Despite widespread interest, progress toward a comprehensive MSI risk prediction model has stagnated. Field-expedient biomechanical screens, such as the Functional Movement Screen (FMS) and the Y-balance test (YBT), have gained popularity due to early work showing promise in predicting injury. However, several investigations call into question the predictive validity of these tools, citing inconsistencies in the associations between scores and injury rates across heterogeneous samples [3,4,5,6,7,8]. Moreover, recent work has pointed to low test precision, tainting the practical utility of these tools [5,6].

Injury prediction is viewed as a complex system, where the impacts of interconnected components evolve in relation to each other and the outcome. Injury prediction methods incorporating a few predictors or domains (i.e., biomechanics only) have not yielded models with strong sensitivity or specificity. As such, several studies have called for multifactorial models that consider the complex interplay between many injury risk factors, rather than just biomechanical determinants [3,4,5,6,7,8]. Popular movement screens do not consider other potentially risk-increasing factors such as age, sex, body mass index (BMI), exercise experience, overall fitness level, injury history, and other sociodemographic determinants such as military service, and socioeconomic status. The extant literature suggests that these factors may confound the association between injury rates and movement screen scores. For example, multiple studies involving the FMS and YBT in athlete populations show differences in the composite and component scores based on sex, injury history, and sport [5,6,9]. Similar studies reported higher BMI and lower fitness levels in association with lower scores and higher MSI risk [5,6]. Factors such as age and sex, and performance-based characteristics such as strength, joint stability, and flexibility may be nested within injury history, which is arguably the strongest predictor of novel or recurrent injury [10,11]. This is exemplified by higher anterior cruciate ligament injury rates in females, whose susceptibility stems from sex-based differences in flexibility, joint stability, and biomechanics [12].

Occupational exposures were also shown to increase the risk of injury and pain warranting consideration in injury risk screens. Several studies indicate broad risk categories for MSIs related to occupation, including shift work, repetitive patterns or positions, high physical exertion, full-day sedentary work, computer work, and sleep problems [13,14,15,16].

Multifactorial injury risk models incorporating modifiable and non-modifiable factors have demonstrated potential. In studies by Rhon et al. and Teyhen et al., significant injury risk factors were identified in military personnel [7,17,18]. Rhon et al. found a combination of factors such as female sex, high BMI, pain during FMS tests or a score of ≤14, and poor fitness test scores linked to injuries [17]. Teyhen and colleagues reported multiple self-reported factors associated with high injury risk, like smoking, prior surgery, musculoskeletal injury history, limited-duty days, and poor performance on fitness tests [7]. A different study by Teyhen et al. showed that multiple individual factors created a sensitive predictive model for time-loss injuries in Army soldiers [18].

In recent years, the identification of several clinical measures of musculoskeletal health has helped to build risk profiles through a better understanding of pain and injury mechanisms. C-reactive protein (CRP) is an inflammatory biomarker that is linked to musculoskeletal pain in association with obesity, work-related stress, and rheumatism [19]. Fibrinogen (FIB), a reactant marker of inflammation, is an established indicator of tissue injury that was implicated as a deserving target for joint degeneration and chronic inflammation [19]. Bone alkaline phosphatase (BAP) is a serum marker of bone formation and was used to study the magnitude of musculoskeletal stress in response to training exertion [20]. Urinary N-telopeptide (NTx) is a marker of bone resorption often used to monitor disease progression in osteoporosis [21]. Although typically concerning inflammatory bowel diseases, H. pylori (HPY) is a bacterium that is associated with osteoporosis, suggesting possible complex interactions between gastrointestinal health and bone loss [22].

Owing to the complex interplay between human behaviors and adaptive physiological processes, ambiguity exists concerning biomarker dynamics in response to musculoskeletal disorders. For example, BAP and NTx responses were shown to be inconsistent despite similar sample characteristics and training regimens [20]. Furthermore, bone turnover markers (BTM) were shown to increase in proportion to fracture risk in some populations but not others [23]. Inflammation plays a complex role in pain perception, bone and muscle metabolism, and chronic disease. For example, in a fibromyalgia cohort, symptoms were worse in those with higher CRP, which was explained mostly by obesity and physical inactivity despite the prevailing theory that myalgia symptoms are not primarily caused by inflammation [24].

Currently, no comprehensive injury risk model is widely accepted. Due to the breadth of measures relevant to MSIs available in the National Health and Nutrition Examination Survey (NHANES), opportunities exist to explore multifactorial injury risk models underpinned by clinically relevant measures. Hence, the primary objective of this study is to investigate multifactorial models in association with injury, pain, physical functioning, pertinent biomarkers, and individual characteristics. Moreover, an endeavor is made to identify confounding variables that might exert an influence on these associations.

## 2. Materials and Methods

Data including sociodemographic variables, self-reported bone/joint injury, pain, functional difficulty (FD) performing activities of daily living (ADLs), physical activity, body composition, work hours, and markers of inflammation and bone turnover were collected from NHANES years 1999 to 2002. These years were chosen because they included joint pain, fitness measures, and other related variables that were not included in later data collection years. Respondents were excluded from analyses if they were unable to engage in muscle-strengthening activities, recreational activities, or tasks around the home. FDs were totaled by recoding the responses ‘some difficulty’ and ‘much difficulty’ as ‘yes’. The number of joints impacted by pain was totaled by adding the number of affected body regions.

NHANES employs a multistage sampling approach to address variations in selection probabilities, non-response rates, and adjustments for reference population proportions. According to NHANES guidance, 4-year mobile examination center weights were used for analyses combining examination and interview data. Skipped-question predictor variables or user-missing values were numerically coded (e.g., ‘0’) or treated as valid where applicable. Data missing completely at random were excluded listwise during analyses. For the case–control analyses, missing values were replaced with the median.

Descriptive population estimates (means and standard errors) were computed from complex design variables (pseudo-PSU and pseudo-stratum) and stratified by sex, injury models, and case–control (CC) groups. Odds ratios for self-reported bone/joint injury were computed for sociodemographic factors, lifestyle factors, injury risk models, and case–control groups via logistic regression. Receiver operating characteristic (ROC) curve analysis was conducted to determine the predictive precision of membership to a risk factor cluster as a classifier for pain and FDs. This was performed on the total sample and by sex.

### Case–Control

To identify potential confounders, we extracted a CC sample using propensity score matching with a caliper of 0.5 (fuzzy matching). Cases were matched with replacement by age, BMI, percent body fat (PBF), frequency of muscle-strengthening activities (MSAs), FDs, pain count, and estimated VO_2_ max (mL/kg/min). The Kolmogorov–Smirnov test was used to determine the normality of covariate distributions (*p*-value < 0.05). Covariate median differences between the CC groups were computed and then imputed into the logistic regression model to identify significant associations. Odds ratios were subsequently adjusted for identified confounders. Lastly, principal component analysis (PCA) was conducted to identify potential risk factor clusters predictive of injury group membership.

## 3. Results

Data from over 21,033 NHANES respondents were included in the analysis. Descriptive population estimates are summarized in the Supplementary Materials. Approximately 3.2% (0.3%) of respondents reported a bone/joint injury causing difficulty or requiring assistance, and 44.1% (0.8%) reported joint pain/aching/stiffness in the past year. Of those reporting joint pain in the past year, 32% (1.1%) reported pain symptoms due to injury. Approximately 15% (1.3%) of those reporting pain symptoms due to injury also reported bone-joint injury causing difficulty. Whereas 84% (1.9%) of those reporting bone/joint injury causing difficulty reported joint pain/aching/stiffness in the past year, 56% (3.4%) reported pain symptoms due to injury.

There was no association between sex and bone/joint injury (Male: OR 1.186 [95% C.I. 0.986–1.426]). There were strong associations for age group (40–49 years: OR 1.96 [95% C.I. 1.30–2.95]; 50–59 years: OR 2.09 [C.I. 1.37–3.17]; 60 and above: OR 4.82 [95% C.I. 3.32–7.01]), family poverty to income ratio (PIR) (Tercile 1: OR 1.88 [95% C.I. 1.23–2.89]), and veteran/military status (OR 1.52 [95% C.I. 1.14–2.01]).

There were significant associations between individual/lifestyle factors and self-reported bone/joint injury (Table 1). Compared to normal BMI, those who were overweight (OR 1.85 [95% C.I. 1.25–2.72]) and obese (OR 2.87 [95% C.I. 2.09–3.96]) had close to double and triple the odds, respectively. Compared to heavy physical work, a physical activity level (PAL) characterized by sitting most of the day was associated with over three times the odds (OR 3.053 [95% C.I. 1.23–7.58]). Muscle-strengthening activities (MSAs) were associated with a 44% (OR 0.563 [95% C.I. 0.40–0.80]) reduction in odds. For those reporting low back pain (LBP) at the time of the survey, the odds were over 2.5 (OR 2.55 [95% C.I. 1.99–3.304]). Increases in pain count, FDs, and total factors increased the odds by 14.6% (OR 1.146 [95% C.I. 1.114–1.178]), 40.5% (OR 1.405 [95% C.I. 1.355–1.475]), and two-fold (OR 2.024 [C.I. 95% 1.867–2.194]), respectively.

### 3.1. Injury Risk Models

Risk models were constructed from the strength of associations from the univariate logistic regression analysis. Model 1 included those with three or more factors. Model 2 included risk factors in Model 1 plus those reporting LBP during the past 3 months, and Model 3 included risk factors in the first two models plus those with more than one region-specific pain point within the past year. Those not selected to an elevated risk group will be referred to as the ‘low-risk group.’

All injury factor models were strongly associated with self-reported bone/joint injury. Model 1 was associated with odds of 2.24 (95% C.I. 1.71–2.92); Model 2 was associated with odds of 2.94 (95% C.I. 2.06–4.18); and Model 3 was associated with odds of 4.04 (95% C.I. 2.88–5.681). When adjusted for age, the association attenuated for Model 1 (OR 0.93 [95% C.I. 0.71–1.21]) but remained for Models 2 (OR 1.56 [95% C.I. 1.11–2.18]) and 3 (OR 2.15 [95% C.I. 1.54–2.99]). Males were more likely to be members of Model 1 (OR 1.103 [95% C.I. 1.02–1.19]) and less likely to be members of Model 3 (OR 0.80 [95% C.I. 0.68–0.94]) compared to females (Table 2).

There were strong associations for the age groups. Odds were highest in the 50–59-year-old group compared to the 20–39-year-old group across all models (Model 1: OR 31.08 [95% C.I. 23.86–40.48]; Model 2: OR 15.85 [C.I. 11.24–22.65] Model 3: OR 20.66 [C.I. 13.64–31.30]) (Table 2). In those with veteran/military status, odds were highest for Model 1 and similar for Models 2 and 3 (Model 1: OR 2.14 [95% C.I. 1.805–2.526]; Model 2: OR 1.48 [95% C.I. 1.215–1.808]; Model 3: OR 1.58 [95% C.I. 1.253–2.00]). A marginal trend increase in odds across risk models was observed for increases in FDs (Model 1: OR 1.28 [95% C.I. 1.25–1.31]; Model 2: OR 1.30 [95% C.I. 1.27–1.33]; Model 3: OR 1.35 [95% C.I. 1.31–1.39]). Compared to PIR Tercile 3, odds for Model 1 membership in Tercile 1 were significantly lower (OR 0.60 [95% C.I. 0.525–0.684]). Increases in CRP, FIB, and HPY significantly increased the odds of membership to all models (Table 2). However, increases in BTM were significantly associated with decreased odds.

### 3.2. Sensitivity Analysis

**Figure 1:**
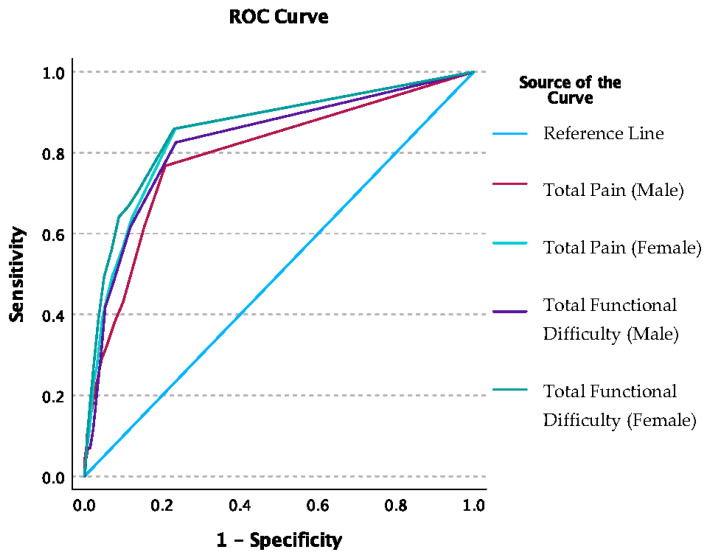
The graph shows the ROC curve sex comparison for total pain and total functional difficulty count classified by selection to the injury factor group: BMI ≥ 30 kg/m^2^, age ≥ 40 years, muscle-strengthening activities = ‘No’, physical activity level = {you sit/he/she sits} during the day and {do/does} not walk about very much, low back pain = ‘Yes’.

Respondents were selected to a risk factor cluster group based on the following criteria: BMI ≥ 30 kg/m^2^, age ≥ 40 years, muscle-strengthening activities = ‘No’, physical activity level = {you sit/he/she sits} during the day and {do/does} not walk about very much, low back pain = ‘Yes’. For the total sample, the area under the ROC curve was 83% (C.I. 80–86%) for the total pain points and 85% (C.I. 82–87%) for the total FD. A total pain count of one or more optimized sensitivity at 70.2% and specificity at 83.4%. A total FD count of one or more optimized sensitivity at 67.4% and specificity at 87.2%. When ROC analysis was conducted by sex, the AUC for total pain points was higher for females at 85% (C.I. 81–88%) compared to males at 79% (C.I. 74–85%) (Figure 1). For FDs, the AUC was again higher for females (86% [C.I. 82–89%]) compared to males (83% [C.I. 78–87%]). A total pain count of one or more optimized sensitivity and specificity for males at 61.6% and 84.6%, respectively; and for females at 75% and 82.2%, respectively. A total FD of one or more optimized sensitivity and specificity for males at 82.6% and 76.5%, respectively; and for females at 86% and 77%, respectively. The AUC difference between sex was not significant for pain (*p*-value = 0.11) or FDs (*p*-value = 0.314).

### 3.3. Case–Control Analysis

The control-matched sample included 467 cases of self-reported bone/joint injury. There were no differences in peak force velocity, physical activity measures, and MSA frequency. Odds for injury group membership were significantly higher for age (24-year increase, OR 3.5 [95% C.I. 2.68–4.68]), BMI (4.5-unit increase, OR 1.53 [95% C.I. 1.29–1.81]), PBF (5.7% increase, OR 1.35 [95% C.I. 1.23–1.48]), total pain count (four-count increase, OR 2.4 [95% C.I. 1.37–4.21]), total FD (five-activity increase, OR 22.62 [95% C.I. 11.52–44.43]), and total factor count (two-factor increase, OR 5.81 [95% C.I. 4.29–7.88]) (Table 3). However, odds were significantly decreased for BAP (one-unit increase, OR 0.971 [95% C.I. 0.956–0.986]) and NTx (63-unit increase, OR 0.963 [95% C.I. 0.941–0.985]). Odds of injury based on an increase in BMI within 1 year prior were equivocal (4.5-unit increase, OR 0.94 [95% C.I. 0.775–1.15]).

When adjusted for age, the associations attenuated for BAP (OR 0.986 [95% C.I. 0.986–1.003]) and NTx (OR 0.986 [95% C.I. 0.964–1.01]) (Table 3). When adjusted for BMI, the odds of injury decreased with increases in BMD (OR 0.965 [C.I. 0.944–0.986]). When adjusted for age, BMI, BAP, NTx, TPF, total FD, total pain, and total factors, all associations attenuated except for total FD (OR 8.57 [95% C.I. 3.80–19.32]).

#### Principal Component Analysis

**Figure 2:**
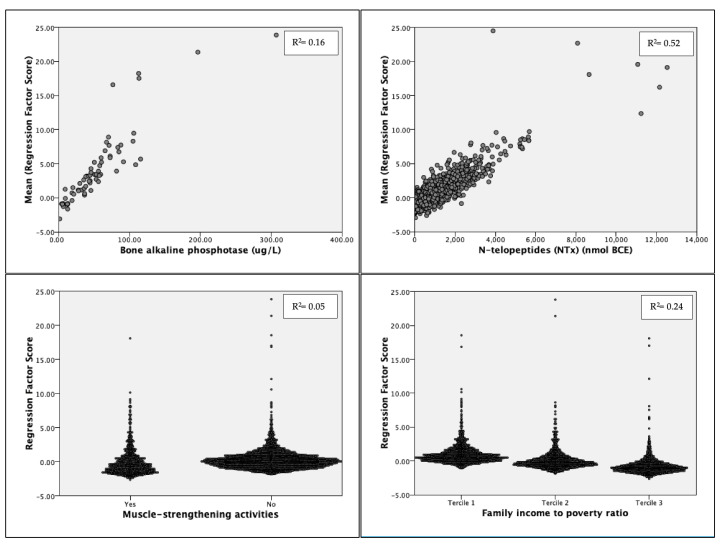
Regression factor score plots for Component 1 predictors. The variables with the strongest loadings are shown (Model R^2^ = 0.97). R^2^ values are linearized and significant at *p* < 0.001. Model SE ± 0.14. Bartlett’s Test of Sphericity: *p* > 0.001.

Predictors age, BMI, NTx, BAP, PAL, LBP, MSAs, veteran/military status, and family PIR maximized the Kaiser–Meyer–Olkin measure at 0.55 and were loaded into four components. The model explained over 57% of the total variance in injury group membership. Component 1 accounted for 17.5%; Component 2: 15.5%; Component 3: 12.34%; and Component 4: 11.83%. In Component 1, NTx (r = 0.604) and BAP (r = 0.603) loaded positively, while MSAs (r = −0.404) and income tercile (r = −0.675) loaded negatively (Figure 2). LBP (r = 0.687) and PAL (r = 0.364) loaded positively, and age (r = −0.716) loaded negatively in Component 2. For Component 3, BMI (r = −0.709) loaded negatively, and PAL (r = 0.668) loaded positively. Lastly, MSA (r = −0.618) and military/veteran status (r = −0.81) loaded negatively in Component 4.

### 3.4. Post Hoc Analyses

**Figure 3:**
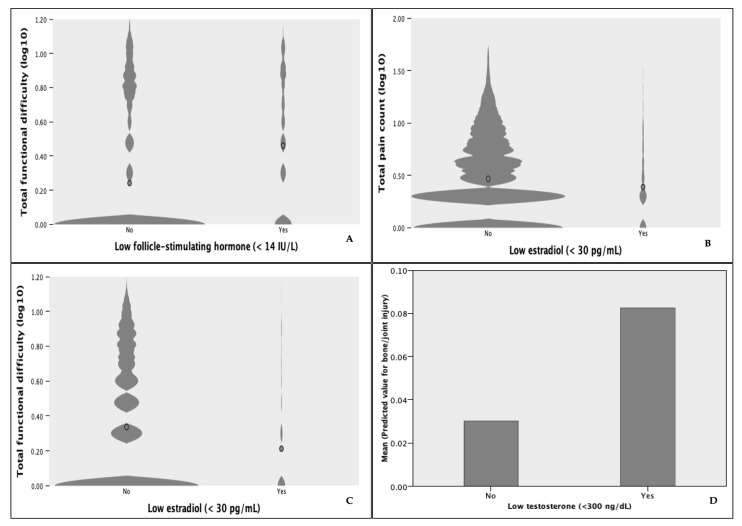
Violin plots of (**A**) total functional difficulty count by FSH level in females (SE ± 0.061) (**B**) total pain count (SE ± 0.03) and (**C**) total functional difficulty count by E2 level in males (SE ± 0.02). (**D**) Mean predicted values for bone/joint injury by T level in males (SE ± 0.004).

Due to the strong loadings of NTx and BAP on principal Components 1 and 2, we performed post hoc analyses to explore the differences in underlying conditions. In the injury group, 16.2% [C.I. 12.9–20.2%] were told they had osteoporosis or brittle bones compared to 4.4% in the control group (OR 4.19 [95% C.I. 1.32–13.33] *p*-value = 0.011). The proportion of respondents receiving treatment for osteoporosis was not significantly different between the groups (OR 0.351 [95% C.I. 0.079–1.552], *p*-value = 0.146). On average, those receiving treatment were older ($\bar{d}$ = 4.94 years; *p*-value < 0.001), and values for BAP ($\bar{d}$ = −0.35; *p*-value < 0.001) and NTx ($\bar{d}$ = −111.57; *p*-value < 0.001) were markedly lower except for higher BAP in the injured reporting treatment compared to the injured receiving no treatment (*p*-value < 0.001). Values for NTx and BAP were higher overall in the injury group regardless of treatment status; however, they were only significant for NTx (*p*-value = 0.012). BTM values were lowest in the control group receiving treatment. In the overall sample, men with total serum testosterone (T) below 300 ng/dl were over 2.8 (95% C.I. 1.057–7.885) times more likely to report bone/joint injury (*p*-value = 0.038) (Figure 3). Increases in pain count decreased the odds of low serum estradiol (E2) (<30 pg/mL (OR 0.96 [95% C.I. 0.919–0.996], *p*-value = 0.03). Increases in FDs also decreased the odds of low E2 (OR 0.91 [95% C.I. 0.862–0.959], *p*-value < 0.001). Similarly, increases in FDs decreased the odds of follicle-stimulating hormone (FSH) below 14 IU/L (indicative of normal estrogen levels) (OR 0.798 [95% C.I. 0.71–0.90]).

## 4. Discussion

In this exploratory analysis, important insights were revealed into the complex nature of musculoskeletal injury prediction. To our knowledge, this is the first comprehensive epidemiological investigation on MSI risk in a U.S. population-representative sample establishing various factor cluster models supported by clinical markers. From a sociodemographic standpoint, increasing age, and lower socioeconomic level (when matched by several covariates) significantly increased the odds of self-reported injury. We also found an over 50% increase in MSI risk for those with a veteran/military background compared to the nonmilitary population. Recent work supports sociodemographic risk factors for MSIs; however, less is known about the causes of increased risk from military service [25,26]. Although active-duty service members tend to be fitter than their age-matched civilian counterparts, veterans have comparatively higher chronic disease rates including musculoskeletal disorders [26]. Exposures common to career military service are certainly culpable, such as regular, repetitive, and sustained physical work and combat-related hazards requiring rapid and intensive physical responses. Likely, the combination of discriminant factors discussed heretofore in addition to service-related exposures explains the increased MSI risk.

Supporting previous studies, our findings showed that lifestyle factors including increasing BMI, sedentary lifestyle, and self-reported LBP substantially increased the odds of self-reported injury while MSAs decreased the odds [25,27]. LBP was highly prevalent in the U.S. population at about 40% and added 70% increased risk in Model 2 compared to Model 1. We did not find an association between bone/joint injury and weekly work hours. Instead, overall PAL was a significant factor, aligning with previous work [15]. In the risk model analysis, we found factor clusters of three or more greatly increased the odds of bone/joint injury and were associated with increased inflammatory markers. When adjusted for age, Model 1 associations attenuated while Models 2 and 3 remained significant, highlighting the effects of non-age-related factors on injury risk. Furthermore, there was an average of over 1.5 injury factors in the low-risk group, emphasizing the importance of using factor clusters to differentiate MSI risk.

A strength of this study was the inclusion of a CC analysis to explore potential confounders. Compared to controls matched on multiple variables, the injury group was older and had elevated BMI, higher TPF, more risk factors, and higher BTMs. Addressing reverse causality, we found no significant association between injury and a recent increase in BMI, suggesting that BMI did not increase because of injury but was a potential causal factor. Only after adjustment for BMI did increases in BMD significantly decrease the odds of injury, indicating that BMI confounded the relationship between BMD and injury. Multiple studies have demonstrated a positive relationship between BMI and BMD [28]. However, our findings support recent work showing a saturation effect of BMI on BMD [28].

Interestingly, we found conflicting results regarding the associations between BTM and bone/joint injury risk. While BAP and NTx were inversely associated with membership to the injury group in the logistic regression, the PCA and post hoc analyses showed a positive relationship. Going beyond basic correlation or regression analysis, PCA attempts to capture maximum variance by creating new uncorrelated components from linear combinations of predictor variables. Instead of eliminating less-predictive collinear variables, such as in stepwise regression, PCA loads combinations of correlated predictors onto components, from the most to the least predictive of the dependent variable. In the aggregate sample, age, and BTMs were predictive of injury but inversely correlated. The PCA accurately reflected positively loaded NTx and BAP with other strongly correlated variables onto Component 1 that were more predictive of injury compared to the combination of variables correlated with age. This explains the strong loading of age on Component 2 and the attenuated odds ratios for BAP and NTx after adjustment for age in the logistic regression. These findings are supported by the observed pattern of BTM values that decline with age but are higher in children and in those at risk for fractures [29].

Another strength of this study was the use of ROC curve analysis to clarify the precision of a multifactorial model to predict pain count and FDs. Test sensitivity and specificity were high, albeit optimized for one or more of each. It is important to note that joint pain and FDs were not exclusively associated with injury. Moreover, the association between pain and injury attenuated after adjustment for other significant predictors, supporting work showing pain as a suboptimal predictor of injury or movement performance [30,31]. Consequently, total joint pain was an unremarkable predictor of bone/joint injury.

Our findings align with work supporting the connection between inflammatory responses and musculoskeletal disorders brought on by metabolic disease processes, which implicates MSIs as sequelae of chronic disease. For instance, elevated markers of inflammation and muscle and bone degradation are often concomitant with metabolic conditions such as menopause, osteoporosis, diabetes, obesity, and cardiovascular disease [21,22,32]. As a direct driver of inflammation and bone loss, fibrinogen may potentiate the differentiation of monocytes into osteoclasts via the receptor activator of the NF-kB ligand (RANKL) pathway [32]. Coincidentally, impaired fibrinolysis was shown to play a role in the pathology of chronic metabolic diseases including those mentioned previously [32]. In a susceptible host, HPY may induce local and systemic inflammatory responses, releasing proinflammatory factors, which further activate osteoclasts resulting in bone resorption [22].

Our investigation corroborated studies reporting associations between hormonal dysregulation, higher BTMs, and injury variables in males and females [33,34]. Stressors such as aging, sleep deprivation, lack of recovery time, excessive workloads, and changes in hormone homeostasis are associated with proinflammatory processes and disruptions in bone metabolism through pathways shared by the immune and bone systems [35]. However, the resulting pathological cascade was shown to vary in different populations for different reasons. Several studies in active participants have shown short-term (hours/days), dose-dependent increases in BTMs in response to novel physical activity [20]. The return to baseline is prolonged with higher relative increases in intensity and duration. Similar studies have also shown long-term (weeks) decreases in BTMs, implicating excessive workloads and inadequate recovery as causal factors [20]. Meanwhile, increased BTMs and low E2 in post-menopausal women were associated with a three-fold increase in fracture risk after adjustment for BMD [20,35]. In men, mounting evidence implicates declining testosterone levels as drivers of bone resorption and decreased BMD, contributing to increased osteoporotic fracture risk [34].

Characterizing the human milieu, our PCA results may have captured clustered risk factors in those with bone/joint injury. Component 1 indicates a pattern related to lack of exercise, socioeconomic disparities, and BTM dysregulation. Component 2 suggests a pattern of higher activity levels and lower back pain in younger individuals. Similarly, Component 3 exhibits a pattern related to higher activity levels and lower BMI. Interestingly, Components 2 and 3 appear to be reflecting inadequate recovery. Lastly, Component 4 displays a pattern possibly related to lack of exercise in civilians compared to those with military/veteran status. Because increases in BTMs appear to be acute in response to PA and decreases are the result of age or chronic stressors, our cross-sectional observations of BTMs appear to reflect habitual behavior such as PA or strength training, non-modifiable factors such as age and socioeconomic level, and underlying chronic disease. This is supported by factor loadings that are consistent with studies showing similar influences of age, BMI, musculoskeletal disorders, mechanical loading, and activity levels on BTMs [21].

### 4.1. Limitations

The most apparent limitations of this study are self-reported data, such as injury and disease status, which may be subject to bias, and the cross-sectional nature of the analysis, which limits causal inference. Moreover, no clinical measures of injury severity were available. Studies have shown that region-specific pain and MSIs differ based on sex, occupation, or previous injury [14,15,25]. Our investigation focused solely on broad injury risk and, therefore, we did not differentiate risk based on a plurality of injury locales or examine differential risk profiles based on sex. Due to retrospective analyses, it is unclear whether ADL difficulty could be used as an injury predictor or whether it is exclusively the result of injury, despite the prevalence of FDs in the control group and association with risk factor models.

### 4.2. Practical and Clinical Considerations

- Pain not due to recent injury is prevalent in the general population and among those with trouble during ADLs. Pain and inflammation are commonly indicative of current MSI but could be caused by previous injury, underlying disease processes, or a combination thereof. Our findings highlight the interdependent nature of chronic disease, pain, and MSIs and emphasize the need to differentiate the causes of pain, which should dictate the process of care. Furthermore, these relationships demonstrate the need for holistic approaches to MSI and chronic disease management.
- Identification of lifestyle risk factor clusters should be prioritized during routine clinical care given their strong associations with injuries, pain, and functional difficulties. Likewise, elevated risk due to demographic factors including veteran or socioeconomic status should be considered in injury prevention strategies. In practice, injury risk could be systematically assessed by capturing accessible information through intake questionnaires, which are commonly used within clinical and non-clinical settings.
- Total FD was found to be an independent predictor of bone/joint injury. Presumably, FD ratings vary according to subjective norms for physical functioning performance and reflect relative decreases in movement competence. This questions the validity of movement screens scored on movement pattern ideals. Moreover, idealized movement criteria would be rendered invalid for movement contexts in which the supposed ideal performance departs from movement strategies deemed functional by the individual. Ratings of FD during ADLs may be useful in building programs that incorporate individualized prescriptions; however, more research is needed to determine the usefulness of FD ratings in identifying high-risk groups.

## 5. Conclusions

The findings from this study support a combination of modifiable and non-modifiable factors in assessing injury risk in the U.S. population. Moreover, these risk factors were validated by clinical markers and by the prevalence of chronic disease in affected groups. Together with other biomarkers, BTMs may offer valuable insights regarding musculoskeletal injury risk as they may reflect age-related physiological processes, overall lifestyle, and underlying disease. However, these findings may require further validation due to conflicting results from different analyses. This would help ensure the reliability and accuracy of BTMs as predictors of injury. Future research should incorporate prospective study design and objective measures of injury, such as clinical assessments, as well as differentiate risk based on injury types, injury locales, and other relevant factors such as sex and disease progression. This would provide a more nuanced understanding of injury patterns and risk factors at the population level. Practitioners concerned with MSI risk should consider a multifactorial pre-screening tool to foster safe and effective physical activity participation and reduce the burden of musculoskeletal disorders.

## Figures and Tables

**Table 1:** The table shows odds ratios for the dependent variable: health problems causing difficulty from bone/joint injury. ^‡^ PIR = family income to poverty level ratio. ^†^ Reference category. * Significant at *p* < 0.01.

Factors	Categories	Odds Ratio	95% Confidence Interval	
			Lower	Upper
BMI categories	Underweight	0.075 *	0.018	0.316
	Normal			
	Overweight	1.846 *	1.252	2.722
	Obese	2.874 *	2.088	3.955
Usually work 35 or more hours per week		0.700	0.435	1.127
Avg level of physical activity each day	{you sit/he/she sits} during the day and {do/does} not walk about very much.	3.053 *	1.229	7.584
	{you stand or walk/he/she stands or walks} about a lot during the day, but {do/does} not have to carry or lift things very often.	2.330	0.970	5.596
	{you/he/she} lift(s) light load or {have/has} to climb stairs or hills often.	1.539	0.715	3.314
	{you/he/she} {do/does} heavy work or {carry/carries} heavy loads ^†^			
Family PIR Tercile ^‡^	1.00	1.881 *	1.226	2.885
	2.00	1.134	0.793	1.622
	3.00 ^†^			
Muscle-strengthening activities	None ^†^	0.563 *	0.396	0.803
Low back pain	None ^†^	2.552 *	1.987	3.304
Veteran/Military Status	No ^†^	1.520 *	1.14	2.01

**Table 2:** The table shows odds ratios for membership to each model by sociodemographic factor or covariate unit increases. ^†^ Reference categories: Age = 20–39 years; PIR = Tercile 3. * Significant at *p* < 0.01. Dependent variables: Model 1: ≥3 lifestyle factors. (Age ≥ 40 yrs., physical activity level = 1 or 2, BMI category ‘overweight’ or ‘obese,’ answer ‘no’ to muscle-strengthening activities). Model 2: Model 1 + low back pain (During the past 3 months], did {you/SP} have low back pain?). Model 3: Model 2 + >1 region-specific pain point injury group.

			Model 1		Model 2			Model 3		
Predictors	Categories ^†^/Units of Change	95% Confidence Interval			95% Confidence Interval			95% Confidence Interval		
		Odds Ratio	Lower	Upper	Odds Ratio	Lower	Upper	Odds Ratio	Lower	Upper
Male sex	Female	1.103 *	1.020	1.193	0.965	0.827	1.125	0.801 *	0.681	0.942
Age group	40–49	23.468 *	17.547	31.387	12.342 *	8.257	18.449	12.728 *	8.163	19.848
	50–59	31.080 *	23.861	40.484	15.951 *	11.235	22.646	20.663 *	13.641	31.298
	60 and above	25.645 *	19.527	33.679	12.771 *	8.408	19.398	16.484 *	10.374	26.194
Veteran/Military Status	Yes	2.135 *	1.805	2.526	1.482 *	1.215	1.808	1.582 *	1.253	1.996
Functional difficulties	1.00	1.28 *	1.245	1.31	1.30 *	1.265	1.334	1.35 *	1.31	1.390
Family PIR Tercile	1.00	0.599 *	0.525	0.684	0.834	0.676	1.029	0.916	0.720	1.165
	2.00	0.919	0.794	1.063	1.046	0.830	1.319	1.065	0.818	1.388
C-reactive protein (mg/dL)	1.00	1.666 *	1.454	1.909	1.390 *	1.277	1.513	1.421 *	1.289	1.566
Fibrinogen (mg/dL)	100.00	1.309 *	1.180	1.451	1.61 *	1.023	1.318	1.229 *	1.070	1.411
Bone alkaline phosphatase (ug/L)	1.00	0.966 *	0.963	0.969	0.973 *	0.969	0.977	0.974 *	0.969	0.978
N-telopeptides (NTx) (nmol BCE)	100.00	0.910 *	0.897	0.924	0.922 *	0.908	0.936	0.919 *	0.902	0.938
Helicobacter pylori (ISR)	1.00	1.352 *	1.281	1.427	1.252 *	1.155	1.357	1.242 *	1.108	1.393

**Table 3:** The table displays odds ratios for injury group membership based on sex and imputed median differences. Cases were matched with controls by age, BMI, PBF, frequency of muscle-strengthening, FDs, pain count, and estimated VO_2_ max (mL/kg/min). ^‡^ ISR = Immune Status Ratio, detected through IgG antibodies. ^†^ Reference category. Mean propensity scores for the injury and control groups were 0.167 and 0.037, respectively. * Significant at *p* <0.01.

					Age-Adjusted				BMI-Adjusted	
	Units of Change (Abs. Med. Diff.)	Odds Ratio	95% Confidence Interval		Odds Ratio	95% Confidence Interval		Odds Ratio	95% Confidence Interval	
			Lower	Upper		Lower	Upper		Lower	Upper
Male	Female ^†^	0.869	0.639	1.183	0.983	0.658	1.467	0.916	0.638	1.314
Age at screening	24.00	3.545 *	2.683	4.684				3.095 *	2.26	4.24
Body mass index (kg/m^2^) Change in BMI from 1 year ago	4.50 4.50	1.528 * 0.943	1.291 0.775	1.81 1.147	1.36 *	1.15	1.61			
Estimated VO_2_ max (ml/kg/min)	2.13	0.961	0.896	1.030	0.983	0.90	1.076			
Total percent fat (DXA)	5.70	1.348 *	1.226	1.482	1.22 *	1.11	1.348			
Total pain count	4.00	2.403 *	1.372	4.207	1.94 *	1.51	3.72	2.17 *	1.242	3.80
Weeks of joint pain due to injury	3.00	1.118 *	1.052	1.188	1.17 *	1.08	1.20	1.15 *	1.06	1.26
Total functional difficulties	5.00	22.621 *	11.517	44.431	13.16 *	6.66	25.99	17.81 *	9.05	35.04
Bone mineral density (g/cm^2^)	0.02	0.492	0.143	1.687	1.01	0.987	1.033	0.965 *	0.944	0.986
Bone alkaline phosphatase (ug/L)	1.00	0.971 *	0.956	0.986	0.986	0.969	1.003	0.973 *	0.955	0.991
C-reactive protein (mg/dL)	0.12	1.024	0.968	1.084	1.003	0.967	1.041	1.003	0.978	1.028
Fibrinogen (mg/dL)	13.00	1.051	0.993	1.113	1.034	0.976	1.096	1.033	0.972	1.097
Helicobacter pylori (ISR) ^‡^	0.15	1.043	1.00	1.088	1.02	0.975	1.07	1.033	0.990	1.078
N-telopeptides (NTx) (nmol BCE)	63.00	0.963 *	0.941	0.985	0.986	0.964	1.01	0.963 *	0.941	0.986
Total factor count	2.00	5.811 *	4.286	7.877	3.907 *	2.70	5.70	5.76 *	4.02	8.26

## Data Availability

The data that support the findings of this study are available from the corresponding author upon reasonable request. These data were derived from the following resources available in the public domain: https://www.cdc.gov/nchs/nhanes/index.htm (accessed on 1 August 2023).

## Supplementary Materials

The following supporting information can be downloaded at: https://www.mdpi.com/article/10.3390/sports12050123/s1.
