# Supplementary material for: Predictive Validity of Multifactorial Injury Risk Models and Associated Clinical Measures in the U.S. Population
Source: Sports (Basel). 2024 Apr 28;12(5):123. doi: 10.3390/sports12050123 (PMC11125903; doi:10.3390/sports12050123)
Supplement: Supplementary file 1 [file sports-12-00123-s001.zip › sports-2952283-supplementary.pdf]

Symptoms begin only because of injury

		Estimate	Standard Error	Unweighted Count
Population Size	Yes	27008823.000	1550881.929	1146
	No	57657161.000	1991156.344	2767
	Total	84665984.000	3030523.368	3913
% of Total	Yes	31.9%	1.1%	1146
	No	68.1%	1.1%	2767
	Total	100.0%	0.0%	3913

Bone/joint injury

		Estimate	Standard Error	Unweighted Count
Population Size	No	269765543.000	7967611.958	19292
	Yes	8886724.000	923183.502	467
	Total	278652267.000	8335243.942	19759
% of Total	No	96.8%	0.3%	19292
	Yes	3.2%	0.3%	467
	Total	100.0%	0.0%	19759

*Symptoms begin only because of injury * Bone/joint injury*

Symptoms begin only because of injury			Bone/joint injury No	Yes	Total
Yes	Population Size	Estimate	22925762.000	4083061.000	27008823.000
		Standard Error	1375676.426	420822.769	1550881.929
	% within Symptoms begin only because of injury	Estimate	84.9%	15.1%	100.0%
		Standard Error	1.3%	1.3%	0.0%
	% within Bone/joint injury	Estimate	29.6%	55.9%	31.9%
		Standard Error	1.1%	3.4%	1.1%
	% of Total	Estimate	27.1%	4.8%	31.9%
		Standard Error	1.0%	0.5%	1.1%
No	Population Size	Estimate	54429499.000	3227662.000	57657161.000
		Standard Error	1899019.431	484468.926	1991156.344
	% within Symptoms begin only because of injury	Estimate	94.4%	5.6%	100.0%
		Standard Error	0.8%	0.8%	0.0%
	% within Bone/joint injury	Estimate	70.4%	44.1%	68.1%
		Standard Error	1.1%	3.4%	1.1%
	% of Total	Estimate	64.3%	3.8%	68.1%
		Standard Error	1.3%	0.5%	1.1%
Total	Population Size	Estimate	77355261.000	7310723.000	84665984.000
		Standard Error	2810915.804	774671.497	3030523.368
	% within Symptoms begin only because of injury	Estimate	91.4%	8.6%	100.0%
		Standard Error	0.8%	0.8%	0.0%
	% within Bone/joint injury	Estimate	100.0%	100.0%	100.0%
		Standard Error	0.0%	0.0%	0.0%
	% of Total	Estimate	91.4%	8.6%	100.0%
		Standard Error	0.8%	0.8%	0.0%

Tests of Independence

		Chi-Square	Adjusted F	df1	df2	Sig.
Symptoms begin only because of injury * Bone/joint injury	Pearson	97.644	61.263	1	29	<.001
	Likelihood Ratio	90.617	56.854	1	29	<.001

The adjusted F is a variant of the second-order Rao-–Scott-–adjusted Cchi-square statistic. Significance is based on the adjusted F and its degrees of freedom.

Descriptive Estimates by Injury Status

	No injury				Bone/joint injury			
			95% Confidence Interval				95% Confidence Interval	
	Estimate	S.E.	Lower	Upper	Estimate	S.E.	Lower	Upper
Age at screening	34.86	0.38	34.06	35.65	56.89	1.20	54.42	59.35
Body mass index (kg/m^2)	25.76	0.12	25.51	26.02	29.94	0.41	29.08	30.79
Total percent fat (DXA)	32.81	0.13	32.54	33.08	36.99	0.51	35.94	38.03
Estimated VO2max (ml/kg/min)	41.10	0.29	40.50	41.70	39.83	3.46	32.75	46.91
Family PIR	2.79	0.061	2.67	2.92	2.35	0.16	2.02	2.68
Total pain count	1.43	0.046	1.33	1.52	6.35	0.54	5.23	7.47
#wks have joint pain symptoms	22.00	1.09	19.76	24.24	37.73	1.57	34.51	40.96
Total functional difficulty	0.81	0.021	0.76	0.85	5.28	0.19	4.88	5.69
# of times past 30 days (tasks around home)	10.31	0.38	9.53	11.09	14.81	1.84	11.03	18.58
Total bone mineral density (g/cm^2)	1.10	0.002	1.09	1.10	1.091	0.0079	1.07	1.10
Bone alkaline phosphatase (ug/L)	25.01	0.49	24.00	26.02	16.45	0.66	15.09	17.82
C-reactive protein(mg/dL)	0.34	0.0075	0.32	0.35	0.53	0.045	0.44	0.62
Fibrinogen (mg/dL)	364.04	2.78	358.34	369.73	383.28	7.35	368.24	398.33
Helicobacter pylori (ISR)	0.81	0.030	0.75	0.884	1.04	0.085	0.86	1.23
N-telopeptides (NTx) (nmol BCE)	715.15	18.68	676.93	753.37	418.68	24.73	368.09	469.26
Average peak force (Newtons)	285.58	2.84	279.77	291.39	244.65	8.13	228.01	261.28
Peak force (Newtons)	364.29	2.86	358.43	370.15	319.14	9.14	300.43	337.85
Peak force velocity degree/seconds	53.87	0.458	52.93	54.81	46.97	2.57	41.69	52.25
Hours worked last week at all jobs	40.71	0.381	39.90	41.53	36.10	2.86	30.00	42.21
How long per day (minutes) (walked or bicycled)	43.85	1.13	41.53	46.16	42.24	5.58	30.82	53.67
How long each time (minutes) (tasks around home)	85.26	1.62	81.93	88.59	88.07	7.62	72.48	103.66
Number of times past 30 days (muscle strengthening activities)	14.73	0.207	14.31	15.15	18.09	3.48	10.96	25.22
Total factor count	2.10	0.024	2.05	2.15	3.74	0.067	3.60	3.87

Table shows weighted mean estimates for the total sample by reported injury status.

Descriptive Estimates by Sex

Gender			Estimate	Standard Error	95% Confidence Interval Lower	95% Confidence Interval Upper	Population Size	Unweighted Count
Male	Mean	Age at Screening	34.44	.392	33.64	35.24	135811155.000	9660
		Body Mass Index (kg/m**2)	25.6527	.11158	25.4245	25.8809	127215057.000	8478
		Total percent fat (DXA)	27.2958	.12224	27.0458	27.5458	116807325.000	7406
		Estimated VO2max (ml/kg/min)	45.0712	.30782	44.4417	45.7008	43111050.000	2822
		Family PIR	2.8700	.06280	2.7416	2.9984	124785377.000	8713
		Total pain count	1.3763	.06282	1.2478	1.5047	135811155.000	9660
		#wks have joint pain symptoms	24.73	1.175	22.33	27.14	15010155.000	611
		Total functional difficulty	.7781	.02802	.7208	.8354	135811155.000	9660
		# of times past 30 days (tasks around home)	9.54	.269	8.99	10.09	67668301.000	3197
		Total Bone Mineral Density (g/cm^2)	1.1364	.00236	1.1316	1.1412	116807325.000	7406
		Bone alkaline phosphatase (ug/L)	28.9237	.58697	27.7232	30.1242	102771142.000	6485
		C-reactive protein(mg/dL)	.2815	.00766	.2659	.2972	110866513.000	7258
		Fibrinogen (mg/dL)	356.53	3.279	349.82	363.24	47229825.000	2533
		Helicobacter pylori (ISR)	.8349	.03003	.7705	.8994	55345636.000	3664
		N-telopeptides (NTx) (nmol BCE)	911.88	30.058	850.40	973.35	94671388.000	5409
		Average peak force (Newtons)	336.8201	5.12957	326.3289	347.3113	26090176.000	1540
		Peak force (Newtons)	440.89	4.953	430.76	451.02	26090176.000	1540
		Peak force velocity degree/seconds	56.0228	.41860	55.1667	56.8789	26090176.000	1540
		Hours worked last week at all jobs	44.26	.395	43.42	45.10	37826994.000	1736
		How long per day (minutes) (walked or bicycled)	47.50	1.773	43.88	51.13	30119388.000	2157

		How long each time (minutes) (tasks around home)	89.90	1.592	86.64	93.16	67580252.000	3193
		Number of times past 30 days (muscle strengthening activities)	15.37	.296	14.76	15.97	39655010.000	2693
		Total factor count	2.1086	.02412	2.0593	2.1579	135811155.000	9660
Female	Mean	Age at Screening	36.63	.464	35.68	37.58	142841112.000	10099
		Body Mass Index (kg/m**2)	26.1219	.15659	25.8017	26.4422	134368342.000	8994
		Total percent fat (DXA)	38.9437	.16979	38.5965	39.2910	111112508.000	6093
		Estimated VO2max (ml/kg/min)	36.8379	.28910	36.2467	37.4292	40170742.000	2693
		Family PIR	2.7043	.06168	2.5782	2.8305	130147364.000	9063
		Total pain count	1.7933	.05535	1.6801	1.9065	142841112.000	10099
		#wks have joint pain symptoms	23.97	1.432	21.04	26.90	11338830.000	509
		Total functional difficulty	1.1207	.02791	1.0636	1.1778	142841112.000	10099
		# of times past 30 days (tasks around home)	11.49	.632	10.20	12.79	62205668.000	2896
		Total Bone Mineral Density (g/cm^2)	1.0654	.00240	1.0605	1.0703	111112508.000	6093
		Bone alkaline phosphatase (ug/L)	20.6216	.54360	19.5098	21.7334	103956036.000	6411
		C-reactive protein(mg/dL)	.4107	.01300	.3841	.4373	114822282.000	7584
		Fibrinogen (mg/dL)	372.59	2.893	366.67	378.51	53048284.000	2533
		Helicobacter pylori (ISR)	.8145	.03488	.7397	.8893	57520476.000	3829
		N-telopeptides (NTx) (nmol BCE)	503.85	21.768	459.33	548.38	97467863.000	5349
		Average peak force (Newtons)	236.5307	2.25055	231.9278	241.1336	29339000.000	1500
		Peak force (Newtons)	292.38	2.522	287.23	297.54	29363036.000	1502
		Peak force velocity degree/seconds	51.3732	.71408	49.9127	52.8336	29363036.000	1502
		Hours worked last week at all jobs	36.13	.547	34.96	37.29	31056195.000	1472

How long per day (minutes) (walked or bicycled)	40.00	1.446	37.04	42.96	29383112.000	2135
How long each time (minutes) (tasks around home)	80.42	2.056	76.21	84.62	62090571.000	2889
Number of times past 30 days (muscle strengthening activities)	14.09	.272	13.54	14.65	31031925.000	2028
Total factor count	2.2018	.02970	2.1411	2.2626	142841112.000	10099

Odds Ratios for Demographic Groups

Categories		Odds Ratio	95% Confidence Interval Lower	Upper
Male sex	Female†	1.186	0.986	1.426
Age group	20-39†	.	.	.
	40-49	1.957*	1.299	2.947
	50-59	2.087*	1.373	3.171
	60 and above	4.824*	3.319	7.010
Education Level - Adults 20+	Less Than 9th Grade†	.	.	.
	9-11th Grade (Includes 12th grade with no diploma)	0.578*	0.353	0.948
	High School Grad/GED or Equivalent	0.530*	0.336	0.836
	Some College or AA degree	0.483*	0.321	0.726
	College Graduate or above	0.353*	0.205	0.607
Race/Ethnicity	Non-Hispanic White†	.	.	.
	Non-Hispanic Black	0.788	0.576	1.077
	Mexican American	0.446*	0.305	0.653
	Other Race - Including Multi-Racial	0.524	0.259	1.061
	Other Hispanic	1.288	0.505	3.285
Family PIR Tercile‡	1.00	1.881*	1.226	2.885
	2.00	1.134	0.793	1.622
	3.00†	.	.	.
Veteran/Military Status	Yes	1.512*	1.136	2.013

Table shows odds ratios for the dependent variable: health problems causing difficulty from bone/joint injury. †Reference categories. ‡PIR = family income to poverty level ratio. *Significant at p<0.01.

'Low Risk' Model Descriptive Estimates

		Estimate	Standard Error	95% Confidence Interval Lower	Upper	Population Size	Unweighted Count
Mean	Age at Screening	29.25	.394	28.44	30.05	210915458.000	16254
	Body Mass Index (kg/m^2)	24.0698	.09344	23.8787	24.2609	194091361.000	13977
	Total percent fat (DXA)	31.1396	.12550	30.8829	31.3962	160615193.000	10019
	Estimated VO2max (ml/kg/min)	41.3171	.27774	40.7491	41.8852	72804180.000	5104
	Family PIR	2.7000	.06412	2.5689	2.8312	193266093.000	14640
	Total pain count	1.2025	.04072	1.1193	1.2858	210915458.000	16254
	#wks have joint pain symptoms	23.02	73.032	85.30	384.04	18559260.000	734
	Total functional difficulty	.6397	.02271	.5933	.6862	210915458.000	16254
	# of times past 30 days (tasks around home)	10.14	.386	9.35	10.93	88591649.000	4242
	Total Bone Mineral Density (g/cm^2)	1.0948	.00227	1.0902	1.0995	160615193.000	10019
	Bone alkaline phosphatase (ug/L)	28.0806	.58757	26.8789	29.2823	150174764.000	9969
	C-reactive protein(mg/dL)	.2882	.00830	.2713	.3052	168922334.000	11899
	Fibrinogen (mg/dL)	356.25	3.417	349.27	363.24	46614164.000	2247
	Helicobacter pylori (ISR)	.7404	.02748	.6814	.7993	82867190.000	5870
	N-telopeptides (NTx) (nmol BCE)	849.85	23.823	801.13	898.58	133679528.000	7719
	Average peak force (Newtons)	266.1716	3.81554	258.3680	273.9753	23295304.000	1259
	Peak force (Newtons)	338.95	4.599	329.55	348.36	23295304.000	1259
	Peak force velocity degree/seconds	53.0039	.71218	51.5473	54.4605	23295304.000	1259
	Hours worked last week at all jobs	55.76	15.902	21.87	89.66	47092471.000	2304
	How long per day (minutes) (walked or bicycled)	43.74	1.825	40.01	47.47	46396902.000	3617
	How long each time (minutes) (tasks around home)	81.86	1.391	79.02	84.71	88416678.000	4233

	Number of times past 30 days (muscle strengthening activities)	14.79	.198	14.39	15.20	70035821.000	4685
	Total factor count	1.5423	.01567	1.5103	1.5744	210915458.000	16254

Tables shows population descriptive estimates for those not selected to models 1, 2, or 3.

Model 1 Descriptive Estimates

		Estimate	Standard Error	95% Confidence Interval Lower	Upper	Population Size	Unweighted Count
Mean	Age at Screening	55.53	.313	54.89	56.17	39129484.000	2094
	Body mass index (kg/m^2)	30.9199	.15265	30.6077	31.2321	38942758.000	2090
	Total percent fat (DXA)	37.0581	.19668	36.6559	37.4604	38799543.000	2081
	Estimated VO2max (ml/kg/min)	38.9464	.82935	37.2502	40.6427	6335380.000	266
	Family PIR	3.1563	.08123	2.9902	3.3225	35498091.000	1865
	Total pain count	1.5355	.07410	1.3839	1.6870	39129484.000	2094
	#wks have joint pain symptoms	23.03	1.888	19.17	26.89	4023901.000	184
	Total functional difficulty	1.4229	.05840	1.3034	1.5423	39129484.000	2094
	# of times past 30 days (tasks around home)	10.79	.608	9.55	12.03	24335268.000	1109
	Bone mineral density (g/cm^2)	1.1214	.00358	1.1141	1.1287	38799543.000	2081
	Bone alkaline phosphatase (ug/L)	15.6448	.27831	15.0756	16.2140	32827983.000	1751
	C-reactive protein(mg/dL)	0.4977	.01890	.4591	.5364	33016958.000	1765
	Fibrinogen (mg/dL)	372.74	3.457	365.67	379.81	31546751.000	1695
	Helicobacter pylori (ISR)	1.0677	.05746	.9445	1.1910	17171411.000	960
	N-telopeptides (NTx) (nmol BCE)	374.93	10.487	353.48	396.38	34175462.000	1832
	Average peak force (Newtons)	302.3642	3.92549	294.3357	310.3928	19188241.000	1100
	Peak force (Newtons)	386.10	4.535	376.82	395.37	19212277.000	1102
	Peak force velocity degree/seconds	54.3349	.63448	53.0373	55.6326	19212277.000	1102

Hours worked last week at all jobs	42.52	.783	40.85	44.19	13269172.000	568
How long per day (minutes) (walked or bicycled)	42.89	2.336	38.11	47.67	8095282.000	424
How long each time (minutes) (tasks around home)	92.63	3.993	84.47	100.80	24335268.000	1109
Number of times past 30 days (muscle strengthening activities)	17.31	3.372	10.42	24.21	346848.000	25
Total factor count	3.5287	.01749	3.4929	3.5645	39129484.000	2094

Table shows population descriptive estimates for Model 1. Model 1= ≥3 individual factors (Age ≥ 40 yrs., physical activity level = 1 or 2, BMI category 'overweight' or 'obese,' answer 'no' to muscle strengthening activities).

Model 2 Descriptive Estimates

		Estimate	Standard Error	95% Confidence Interval Lower	95% Confidence Interval Upper	Population Size	Unweighted Count
Mean	Age at Screening	54.77	.526	53.70	55.85	28607325.000	1411
	Body Mass Index (kg/m^2)	31.4376	.25941	30.9071	31.9682	28549280.000	1405
	Total percent fat (DXA)	37.7533	.29791	37.1440	38.3625	28505097.000	1399
	Estimated VO2max (ml/kg/min)	40.5765	.93825	38.6576	42.4955	4142232.000	145
	Family PIR	4.5216	.19724	4.1182	4.9250	28607325.000	1411
	Total pain count	2.9129	.07607	2.7573	3.0685	26168557.000	1271
	#wks have joint pain symptoms	31.78	1.849	28.00	35.56	4159578.000	217
	Total functional difficulty	2.6267	.10712	2.4076	2.8458	28607325.000	1411
	# of times past 30 days (tasks around home)	11.75	.728	10.26	13.24	16947052.000	742
	Total Bone Mineral Density (g/cm^2)	1.1144	.00447	1.1052	1.1235	28505097.000	1399
	Bone alkaline phosphatase (ug/L)	16.2564	.33859	15.5639	16.9489	23724431.000	1176
	C-reactive protein(mg/dL)	0.5577	.03275	.4907	.6247	23749503.000	1178
	Fibrinogen (mg/dL)	372.51	3.603	365.14	379.88	22117194.000	1124
	Helicobacter pylori (ISR)	1.0427	.04704	.9418	1.1436	12827511.000	663
	N-telopeptides (NTx) (nmol BCE)	371.32	12.207	346.35	396.29	24284261.000	1207
	Average peak force (Newtons)	287.7328	4.69503	278.1304	297.3352	12945631.000	681
	Peak force (Newtons)	368.80	5.007	358.56	379.04	12945631.000	681
	Peak force velocity degree/seconds	53.4140	1.05263	51.2611	55.5668	12945631.000	681
	Hours worked last week at all jobs	42.55	1.015	40.38	44.71	8531278.000	337
	How long per day (minutes) (walked or bicycled)	45.81	5.208	35.16	56.47	5010316.000	251
	How long each time (minutes) (tasks around home)	93.16	3.996	84.99	101.33	16918877.000	740

Number of times past 30 days (muscle strengthening activities)	14.78	2.225	10.23	19.34	304266.000	11
Total factor count	4.8070	.01601	4.7743	4.8397	28607325.000	1411

Table shows population descriptive estimates for Model 2. Model 2= Model 1 + low back pain (During the past 3 months], did {you/SP} have low back pain?).

Model 3 Descriptive Estimates

		Estimate	Standard Error	95% Confidence Interval Lower	95% Confidence Interval Upper	Population Size	Unweighted Count
Mean	Age at Screening	55.80	.606	54.56	57.03	21442668.000	1033
	Body Mass Index (kg/m^2)	31.8292	.26353	31.2902	32.3681	21384623.000	1027
	Total percent fat (DXA)	38.7077	.23375	38.2296	39.1858	21340440.000	1021
	Estimated VO2max (ml/kg/min)	39.8928	1.16317	37.5139	42.2718	2095033.000	68
	Family PIR	2.8690	.08374	2.6978	3.0403	19717072.000	937
	Total pain count	6.0325	.23979	5.5420	6.5229	21442668.000	1033
	#wks have joint pain symptoms	31.92	1.837	28.16	35.67	4140312.000	215
	Total functional difficulty	3.1526	.13469	2.8771	3.4281	21442668.000	1033
	# of times past 30 days (tasks around home)	11.82	.876	10.03	13.61	12682795.000	560
	Total Bone Mineral Density (g/cm^2)	1.1079	.00360	1.1005	1.1153	21340440.000	1021
	Bone alkaline phosphatase (ug/L)	16.2457	.39134	15.4453	17.0461	17489803.000	843
	C-reactive protein(mg/dL)	.6102	.03969	.5290	.6914	17498019.000	844
	Fibrinogen (mg/dL)	376.26	4.093	367.89	384.63	16599731.000	811
	Helicobacter pylori (ISR)	1.0445	.07003	.8943	1.1947	9172443.000	459
	N-telopeptides (NTx) (nmol BCE)	365.15	15.240	333.98	396.32	18008699.000	870
	Average peak force (Newtons)	284.2546	5.39662	273.2172	295.2919	10202440.000	506
	Peak force (Newtons)	364.34	5.254	353.60	375.09	10202440.000	506
	Peak force velocity degree/seconds	52.7408	1.25778	50.1683	55.3132	10202440.000	506
	Hours worked last week at all jobs	42.09	1.104	39.74	44.45	6336435.000	247
	How long per day (minutes) (walked or bicycled)	48.55	7.283	33.66	63.45	3635455.000	174
	How long each time (minutes) (tasks around home)	95.52	4.803	85.70	105.35	12682795.000	560

Number of times past 30 days (muscle strengthening activities)	14.52	2.932	8.52	20.52	224902.000	7
Total factor count	5.0489	.00780	5.0329	5.0648	21442668.000	1033

Table shows population descriptive estimates for Model 3. Model 3= Model 2 + > 1 region-specific pain point

Model Regression

				Age-Adjusted		
		95% Confidence Interval			95% Confidence Interval	
	Odds Ratio	Lower	Upper	Odds Ratio	Lower	Upper
Model 1	2.237*	1.714	2.920	0.928	0.713	1.208
Model 2	2.936*	2.064	4.178	1.557*	1.111	2.182
Model 3	4.043*	2.877	5.681	2.145*	1.540	2.987

Table shows odds ratios for membership to each model. Dependent variable: health problems causing difficulty from bone/joint injury. *Significant at $p<0.01$. Model 1: ≥3 lifestyle factors. (Age ≥ 40 yrs., physical activity level = 1 or 2, BMI category 'overweight' or 'obese,' answer 'no' to muscle-strengthening activities)
Model 2: Model 1 + low back pain (During the past 3 months], did {you/SP} have low back pain?)
Model 3: Model 2 + > 1 region-specific pain point

ROC Curves of Pain and Functional Difficulty Count by Selection to At-Risk Group

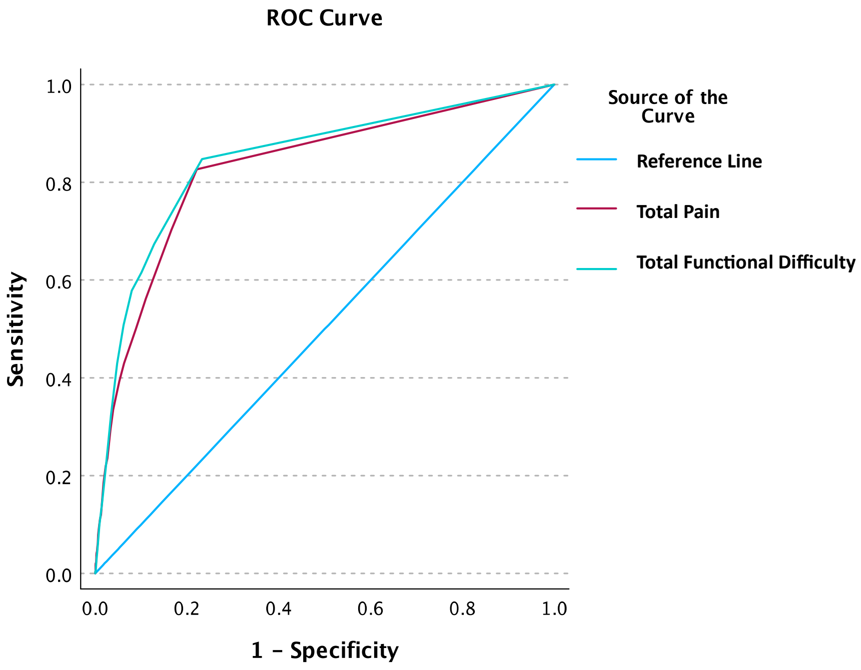

Graph shows ROC curve for total pain and total functional difficulty in respondents selected for the following injury factors: BMI ≥ 30 kg/m^2, age ≥ 40 years, muscle strengthening activities = 'No,' physical activity level = {you sit/he/she sits} during the day and {do/does} not walk about very much, low back pain = 'Yes.'

ROC Classifier Evaluation Metrics (for Figure 1)

Test Result Variable(s)	Gini Index	K-S Statistics	
		Max K-S	Cutoff
Total pain count	.657	.606	.5000
Total functional difficulty	.691	.614	.5000

Table displays inequality index (Gini), distribution comparison, and cut-off values for the classifier (selected to injury factor group).

ROC Classifier Evaluation Metrics (for Figure 2)

Test Result Variable(s)	Gender	Gini Index	K-S Statistics	
			Max K-S	Cutoff
Total pain count	= Male	.588	.560	.5000
	= Female	.690	.625	.5000
Total functional difficulty	= Male	.651	.590	.5000
	= Female	.712	.629	.5000

Table displays inequality index (Gini), distribution comparison, and cut-off values for the classifier (selected to injury factor group) by sex.

Independent-Group Area Difference Under the ROC Curve (for Figure 2)

Test Result Variable(s)	Asymptotic		AUC Difference	Std. Error Difference	Asymptotic 95% Confidence Interval	
	z	Sig. (2-tail)			Lower Bound	Upper Bound
Total pain count	-1.594	.111	-.051	.032	-.114	.012
Total functional difficulty	-1.007	.314	-.031	.030	-.090	.029

Note: Table displays Z-test statistics for sex group differences in area under the ROC curve for injury factor group selection as a classifier for pain and functional difficulty count.

Area Under the ROC Curve

Test Result Variable(s) Unweighted N=242	Area	Std. Error	Asymptotic Sig.	Asymptotic 95% Confidence Interval	
				Lower Bound	Upper Bound
Total pain count	.829	.015	.000	.799	.858
Total functional difficulty	.845	.014	.000	.817	.874

Graph/table shows ROC curve for total pain and total functional difficulty in respondents selected for the following injury factors: BMI ≥ 30 kg/m^2, age ≥ 40 years, muscle strengthening activities = 'No,' physical activity level = {you sit/he/she sits} during the day and {do/does} not walk about very much, low back pain = 'Yes.'

Area Under the ROC Curve by Sex

Test Result Variable(s)	Sex	Area	Std. Error	Asymptotic Sig.	Asymptotic 95% Confidence Interval	
					Lower Bound	Upper Bound
Total pain count	Male (n=86)	.794	.027	.000	.740	.847
	Female (n=156)	.845	.017	.000	.811	.879
Total functional difficulty	Male (n=86)	.825	.025	.000	.776	.874
	Female (n=156)	.856	.017	.000	.822	.890

Note: Graph/table shows ROC curve comparison by sex for total pain and total functional difficulty selected for the following injury factors: BMI ≥ 30 kg/m^2, age ≥ 40 years, muscle strengthening activities = 'No,' physical activity level = {you sit/he/she sits} during the day and {do/does} not walk about very much, low back pain = 'Yes.'

Independent-Group Area Difference Under the ROC Curve

Test Result Variable(s)	Asymptotic z	Asymptotic Sig. (2-tail)	AUC Difference	Std. Error Difference	Asymptotic 95% Confidence Interval	
					Lower Bound	Upper Bound
Total pain count	-1.594	.111	-.051	.032	-.114	.012
Total functional difficulty	-1.007	.314	-.031	.030	-.090	.029

Case-Control Covariate Differences

Health problems causing difficulty from bone/joint injury count (N=934)	Control (n=467)		Injury (n=467)		
	Median	IQR	Median	IQR	Median Difference
Age at screening	33.00	35	57.00	27	24.00
Body mass index (kg/m^2)	24.33	9.77	28.8300	8.68	4.50
Total percent fat (DXA)	32.4000	13.90	38.1000	14.10	5.70
Estimated VO2max (ml/kg/min)	40.3300	11.29	38.20	14.02	-2.13
Family PIR	2.4000	3.20	1.85	2.95	-0.55
Total pain count	0.0000	1.00	4.00	7.00	4.00
#wks have joint pain symptoms	0.00	1.00	4.00	52.00	3.00
Total functional difficulty	0.0000	1.00	5.00	5.00	5.00
Total Bone Mineral Density (g/cm^2)	1.112	0.18	1.092	0.19	-0.02

Bone alkaline phosphatase (ug/L)	16.20	9.60	15.20	8.50	-1.00
C-reactive protein (mg/dL)	0.1700	0.35	0.29	0.47	0.12
Fibrinogen (mg/dL)	356.00	97.00	369.00	91.00	13.00
Helicobacter pylori (ISR)	0.27	1.03	0.4200	1.72	0.15
N-telopeptides (NTx) (nmol BCE)	381.00	600.00	318.00	348.00	-63.00
Average peak force (Newtons)	254.80	134.60	238.50	112.30	-16.30
Peak force (Newtons)	316.00	173.00	313.00	169.00	-3.00
Peak force velocity (degree/seconds)	60.75	1.00	60.7500	2.75	0.00
Hours worked last week at all jobs	40.00	18.00	36.00	20.00	-4.00
Number of times past 30 days (tasks around home)	3.00	9.00	3.00	9.00	0.00

Number of times past 30 days (walked or bicycled)	0.00	1.00	0.00	2.00	0.00
How long per day (minutes) (walked or bicycled)	0.00	20.00	0.00	30.00	0.00
How long each time (minutes) (tasks around home)	30.00	60.00	20.00	90.00	-10.00
Number of times past 30 days (muscle strengthening activities)	0.00	9.00	0.00	8.00	0.00
Total factor count	2.0000	3.00	4.0000	1.00	2.00

Table shows covariate values and median differences between the case-control groups.

Adjusted Odds Ratios for Selection to the Injury Group

	Units of Change	Odds Ratio	95% Confidence Interval	
			Lower	Upper
Age at screening	24.00	1.16	0.673	1.995
Body mass index (kg/m^2)	4.50	1.01	0.735	1.39
Bone alkaline phosphatase (ug/L)	1.00	0.998	0.967	1.03
Total percent fat (DXA)	5.70	1.04	0.813	1.33
Total pain count	4.00	1.22	0.731	2.05
Total functional difficulty	5.00	8.57*	3.80	19.32
N-telopeptides (NTx) (nmol BCE)	63.00	0.99	0.955	1.027
Total factor count	2.00	1.42	0.748	2.70

Dependent Variable: injury group. Odds ratios were adjusted for age, BMI, BAP, NTx, TPF, total functional difficulty, total pain, and total factors. Total factors included age ≥ 40 year, BMI ≥ 25 kg/m^2, PAL = {you sit/he/she sits} during the day and {do/does} not walk about very much OR {you stand or walk/he/she stands or walks} about a lot during the day, but {do/does}not have to carry or lift things very often, no muscle strengthening activities, veteran/military status, and low back pain. *Significant at $p<0.01$.

Principal Component Structure Matrix

	Component			
	1	2	3	4
Body mass index (kg/m^2)	-0.038	0.321	-0.709	0.076
Age at screening	0.071	-0.716	-0.191	0.243
Bone alkaline phosphatase (ug/L)	0.603	-0.205	-0.002	0.039
N-telopeptides (NTx) (nmol BCE)	0.604	0.336	0.175	-0.074
Avg level of physical activity each day	-0.144	0.327	0.668	0.083
Low back pain	0.090	0.687	-0.195	0.104
Muscle strengthening activities	-0.404	0.014	0.373	-0.618
Veteran/Military status	0.198	0.077	-0.211	-0.810
Family income to poverty ratio tercile	-0.675	-0.035	0.250	-0.011

Only cases for which ‘Injury=Yes’ are used in the analysis phase. KMO = 0.55. Bartlett's test of sphericity: $p < 0.001$. Rotation method: Oblimin with Kaiser Normalization.

Odds Ratios for Bone/Joint Injury by Pain Count, Functional Difficulty Count, and Total Injury Factors

		95% Confidence Interval	
Test Variables	Odds Ratio	Lower	Upper
Total pain	1.146*	1.114	1.178
Total functional difficulty	1.405*	1.355	1.457

Total factors	2.024*	1.867	2.194

Dependent variable: health problems causing difficulty from bone/joint injury. Total injury factors included age ≥ 40 years, BMI ≥ 25 kg/m^2, PAL = {you sit/he/she sits} during the day and {do/does} not walk about very much OR {you stand or walk/he/she stands or walks} about a lot during the day, but {do/does}not have to carry or lift things very often, no muscle strengthening activities, veteran/military status, and low back pain.*Significant at p<0.01.

Underlying Bone Condition by Case-Control Group

Ever told had osteoporosis/brittle bones				Case control group: Control	Injury	Total
No	Population Size	Estimate		4417414.000	7364286.000	11781700.000
		Standard Error		396438.433	742003.472	912308.000
		95% Confidence Interval	Lower	3606606.366	5846718.505	9915820.636
			Upper	5228221.634	8881853.495	13647579.364
		Unweighted Count		206	395	601
	% within Ever told had osteoporosis/brittle bones	Estimate		37.5%	62.5%	100.0%
		Standard Error		2.8%	2.8%	0.0%
		95% Confidence Interval	Lower	31.9%	56.6%	100.0%
			Upper	43.4%	68.1%	100.0%
		Unweighted Count		206	395	601
	% within case control group	Estimate		95.6%	83.8%	87.9%
		Standard Error		2.3%	1.8%	1.4%
		95% Confidence Interval	Lower	87.8%	79.8%	84.6%
			Upper	98.5%	87.1%	90.5%
		Unweighted Count		206	395	601
	% of Total	Estimate		32.9%	54.9%	87.9%
		Standard Error		2.7%	2.3%	1.4%
		95% Confidence Interval	Lower	27.7%	50.2%	84.6%
			Upper	38.7%	59.6%	90.5%
		Unweighted Count		206	395	601
Yes	Population Size	Estimate		203886.000	1425338.000	1629224.000
		Standard Error		110013.343	258392.258	269218.731
		95% Confidence Interval	Lower	-21116.550	896866.494	1078609.872
			Upper	428888.550	1953809.506	2179838.128

		Unweighted Count		7	66	73
	% within Ever told had osteoporosis/brittle bones	Estimate		12.5%	87.5%	100.0%
		Standard Error		6.4%	6.4%	0.0%
		95% Confidence Interval	Lower	4.1%	67.7%	100.0%
			Upper	32.3%	95.9%	100.0%
		Unweighted Count		7	66	73
	% within case control group	Estimate		4.4%	16.2%	12.1%
		Standard Error		2.3%	1.8%	1.4%
		95% Confidence Interval	Lower	1.5%	12.9%	9.5%
			Upper	12.2%	20.2%	15.4%
		Unweighted Count		7	66	73
	% of Total	Estimate		1.5%	10.6%	12.1%
		Standard Error		0.8%	1.4%	1.4%
		95% Confidence Interval	Lower	0.5%	8.1%	9.5%
			Upper	4.5%	13.9%	15.4%
		Unweighted Count		7	66	73
Total	Population Size	Estimate		4621300.000	8789624.000	13410924.000
		Standard Error		423558.419	944052.659	1095939.198
		95% Confidence Interval	Lower	3755025.767	6858819.517	11169476.666
			Upper	5487574.233	10720428.483	15652371.334
		Unweighted Count		213	461	674
	% within Ever told had osteoporosis/brittle bones	Estimate		34.5%	65.5%	100.0%
		Standard Error		2.9%	2.9%	0.0%
		95% Confidence Interval	Lower	28.8%	59.3%	100.0%
			Upper	40.7%	71.2%	100.0%
		Unweighted Count		213	461	674
	% within case control group	Estimate		100.0%	100.0%	100.0%
		Standard Error		0.0%	0.0%	0.0%
		95% Confidence Interval	Lower	100.0%	100.0%	100.0%
			Upper	100.0%	100.0%	100.0%
		Unweighted Count		213	461	674
	% of Total	Estimate		34.5%	65.5%	100.0%
		Standard Error		2.9%	2.9%	0.0%
		95% Confidence Interval	Lower	28.8%	59.3%	100.0%
			Upper	40.7%	71.2%	100.0%

	Unweighted Count	213	461	674

Tests of Independence

		Chi-Square	Adjusted F	df1	df2	Sig.
Ever told had osteoporosis/brittle bones * case control group	Pearson	19.874	7.385	1	29	.011
	Likelihood Ratio	23.001	8.547	1	29	.007

The adjusted F is a variant of the second-order Rao-Scott adjusted chi-square statistic. Significance is based on the adjusted F and its degrees of freedom.

Measures of Association

			95% Confidence Interval	
		Estimate	Lower	Upper
Ever told had osteoporosis/brittle bones * case control group	Odds Ratio	4.193	1.320	13.327

Statistics are computed only for 2-by-2 tables with all cells observed.

Descriptive Estimates by Treatment Group

Ever treated for osteoporosis			Estimate	Standard Error	95% Confidence Interval		Unweighted Count
					Lower	Upper	
No	Mean	Age at Screening	61.57	1.667	58.16	64.99	123
		Bone alkaline phosphatase (ug/L)	15.9278	1.00187	13.8721	17.9835	94
		N-telopeptides (NTx) (nmol BCE)	397.85	51.068	293.06	502.63	100
Yes	Mean	Age at Screening	66.51	1.292	63.86	69.16	302
		Bone alkaline phosphatase (ug/L)	15.5739	.74029	14.0550	17.0929	226
		N-telopeptides (NTx) (nmol BCE)	286.28	23.268	238.53	334.02	232

Mean Differences Between Case-Control Groups by Treatment Status

Case control group		Ever treated for osteoporosis	N	Mean	Std. Deviation	Std. Error Mean	p-value
Control	Age at Screening	No	33398	79.00	.000	.000	
		Yes	170488	62.95	16.863	.041	<0.001
	Bone alkaline phosphatase (ug/L)	No	33398	15.0000	.00000	.00000	
		Yes	167141	11.6269	3.87592	.00948	<0.001
	N-telopeptides (NTx) (nmol BCE)	No	33398	391.00	.000	.000	
		Yes	107972	315.44	286.382	.872	<0.001
Injury	Age at Screening	No	508802	62.89	15.217	.021	
		Yes	910556	64.23	19.382	.020	<0.001
	Bone alkaline phosphatase (ug/L)	No	427694	15.2036	4.07621	.00623	
		Yes	546565	19.4637	7.72564	.01045	<0.001
	N-telopeptides (NTx) (nmol BCE)	No	427694	575.14	556.759	.851	
		Yes	521207	392.85	346.420	.480	<0.001

*Ever treated for osteoporosis * case control group*

Ever treated for osteoporosis				case control group: control	injury	Total
No	Population Size	Estimate		33398.000	508802.000	542200.000
		Standard Error		33398.000	133875.970	143122.329
		95% Confidence Interval	Lower	-34908.580	234994.899	249481.970
			Upper	101704.580	782609.101	834918.030
		Unweighted Count		1	20	21
		Estimate		6.2%	93.8%	100.0%

	% within Ever treated for osteoporosis	Standard Error		5.7%	5.7%	0.0%
		95% Confidence Interval	Lower	0.9%	66.7%	100.0%
			Upper	33.3%	99.1%	100.0%
		Unweighted Count		1	20	21
	% within case control group	Estimate		16.4%	35.8%	33.4%
		Standard Error		9.7%	6.2%	5.5%
		95% Confidence Interval	Lower	4.4%	24.3%	23.2%
			Upper	45.4%	49.2%	45.4%
		Unweighted Count		1	20	21
	% of Total	Estimate		2.1%	31.3%	33.4%
		Standard Error		1.9%	5.5%	5.5%
		95% Confidence Interval	Lower	0.3%	21.3%	23.2%
			Upper	13.1%	43.6%	45.4%
		Unweighted Count		1	20	21
Yes	Population Size	Estimate		170488.000	910556.000	1081044.000
		Standard Error		82532.966	179951.559	176963.152
		95% Confidence Interval	Lower	1689.132	542513.737	719113.716
			Upper	339286.868	1278598.263	1442974.284
		Unweighted Count		6	45	51
	% within Ever treated for osteoporosis	Estimate		15.8%	84.2%	100.0%
		Standard Error		7.6%	7.6%	0.0%
		95% Confidence Interval	Lower	5.5%	62.5%	100.0%
			Upper	37.5%	94.5%	100.0%
		Unweighted Count		6	45	51
	% within case control group	Estimate		83.6%	64.2%	66.6%
		Standard Error		9.7%	6.2%	5.5%
		95% Confidence Interval	Lower	54.6%	50.8%	54.6%
			Upper	95.6%	75.7%	76.8%
		Unweighted Count		6	45	51
	% of Total	Estimate		10.5%	56.1%	66.6%
		Standard Error		5.0%	7.4%	5.5%
		95% Confidence Interval	Lower	3.8%	40.9%	54.6%
			Upper	25.7%	70.2%	76.8%
		Unweighted Count		6	45	51
Total	Population Size	Estimate		203886.000	1419358.000	1623244.000

	Standard Error		110013.343	258323.051	269077.934
	95% Confidence Interval	Lower	-21116.550	891028.039	1072917.834
		Upper	428888.550	1947687.961	2173570.166
	Unweighted Count		7	65	72
% within Ever treated for osteoporosis	Estimate		12.6%	87.4%	100.0%
	Standard Error		6.5%	6.5%	0.0%
	95% Confidence Interval	Lower	4.1%	67.6%	100.0%
		Upper	32.4%	95.9%	100.0%
	Unweighted Count		7	65	72
% within case control group	Estimate		100.0%	100.0%	100.0%
	Standard Error		0.0%	0.0%	0.0%
	95% Confidence Interval	Lower	100.0%	100.0%	100.0%
		Upper	100.0%	100.0%	100.0%
	Unweighted Count		7	65	72
% of Total	Estimate		12.6%	87.4%	100.0%
	Standard Error		6.5%	6.5%	0.0%
	95% Confidence Interval	Lower	4.1%	67.6%	100.0%
		Upper	32.4%	95.9%	100.0%
	Unweighted Count		7	65	72

Tests of Independence

		Chi-Square	Adjusted F	df1	df2	Sig.
Ever treated for osteoporosis * case control group	Pearson	1.347	2.231	1	29	.146
	Likelihood Ratio	1.499	2.481	1	29	.126

The adjusted F is a variant of the second-order Rao-Scott adjusted chi-square statistic. Significance is based on the adjusted F and its degrees of freedom.

Measures of Association

		Estimate	95% Confidence Interval Lower	Upper
Ever treated for osteoporosis * case control group	Odds Ratio	.351	.079	1.552

Statistics are computed only for 2-by-2 tables with all cells observed.

Odds Ratio for Functional Difficulty Count by E2 Level

Units of Change		LowE2	Odds Ratio	95% Confidence Interval Lower	Upper
Total functional difficulty	1.000	Yes	.909	.862	.959

Dependent Variable: LOWE2 (reference category = No)

Odds Ratio for Pain Count by E2 Level

Units of Change		LowE2	Odds Ratio	95% Confidence Interval Lower	Upper
Total pain count	1.000	Yes	.957	.919	.996

Dependent Variable: LOWE2 (reference category = No)

Odds Ratio for Functional Difficulty Count by FSH Level

Units of Change		Low follicle stimulating hormone (< 14 IU/L)	Odds Ratio	95% Confidence Interval Lower	Upper
Total functional difficulty	1.000	Yes	.798	.709	.898

Dependent Variable: Low follicle stimulating hormone (< 14 IU/L) (reference category = No)
